# Development of a data-driven case-mix adjustment model for comparison of hospital performance in hip fracture care

**DOI:** 10.1007/s11657-022-01094-w

**Published:** 2022-04-27

**Authors:** Franka S. Würdemann, Arthur K. E. Elfrink, Janneke A. Wilschut, Crispijn L. van den Brand, Inger B. Schipper, Johannes H. Hegeman

**Affiliations:** 1 Dutch Institute for Clinical Auditing, Scientific Bureau, Rijnsburgerweg 10, AA 2333 Leiden, The Netherlands; 2 Department of Traumasurgery, Leiden University Medical Center, Leiden Albinusdreef 2, Leiden, 2333 ZA The Netherlands; 3 Department of Surgery, University Medical Center, Groningen Hanzeplein 1, GZ 9713 Groningen, The Netherlands; 4 Department of Traumasurgery, Ziekenhuisgroep Twente, Zilvermeeuw 1, Almelo, 7609 PP The Netherlands

**Keywords:** Hip fractures, Database, Registry, Case-mix correction, Case-mix factors, Confounders, Hospital comparison, Mortality, Outcomes, Quality of care

## Abstract

**Summary:**

To compare hospitals’ hip fracture patient mortality in a quality of care registry, correction for patient characteristics is needed. This study evaluates in 39,374 patients which characteristics are associated with 30 and 90-day mortality, and showed how using these characteristics in a case mix-model changes hospital comparisons within the Netherlands.

**Purpose:**

Mortality rates after hip fracture surgery are considerable and may be influenced by patient characteristics. This study aims to evaluate hospital variation regarding patient demographics and disease burden, to develop a case-mix adjustment model to analyse differences in hip fracture patients’ mortality to calculate case-mix adjusted hospital-specific mortality rates.

**Methods:**

Data were derived from 64 hospitals participating in the Dutch Hip Fracture Audit (DHFA). Adult hip fracture patients registered in 2017–2019 were included. Variation of case-mix factors between hospitals was analysed, and the association between case-mix factors and mortality at 30 and 90 days was determined through regression models.

**Results:**

There were 39,374 patients included. Significant variation in case-mix factors amongst hospitals was found for age ≥ 80 (range 25.8–72.1% *p* < 0.001), male gender (12.0–52.9% *p* < 0.001), nursing home residents (42.0–57.9% *p* < 0.001), pre-fracture mobility aid use (9.9–86.7% *p* < 0,001), daily living dependency (27.5–96.5% *p* < 0,001), ASA-class ≥ 3 (25.8–83.3% *p* < 0.001), dementia (3.6–28.6% *p* < 0.001), osteoporosis (0.0–57.1% *p* < 0.001), risk of malnutrition (0.0–29.2% *p* < 0.001) and fracture types (all *p* < 0.001). All factors were associated with 30- and 90-day mortality. Eight hospitals showed higher and six showed lower 30-day mortality than expected based on their case-mix. Six hospitals showed higher and seven lower 90-day mortality than expected. The specific outlier hospitals changed when correcting for case-mix factors.

**Conclusions:**

Dutch hospitals show significant case-mix variation regarding hip fracture patients. Case-mix adjustment is a prerequisite when comparing hospitals’ 30-day and 90-day hip fracture patients’ mortality. Adjusted mortality may serve as a starting point for improving hip fracture care.

**Supplementary Information:**

The online version contains supplementary material available at 10.1007/s11657-022-01094-w.

## Introduction

In the Netherlands, approximately 17,500 patients with hip fractures are treated every year [1]. These patients generally show high morbidity and mortality rates. With increasing incidence of hip fractures due to the ageing population and longer life expectancies, the care of these patients will become an even greater challenge for health care providers and the society as a whole [2, 3].

Hip fracture audits have been implemented in several countries and their impact on improving the quality of hip fracture care is growing [4]. The use of quality indicators in audits is widely accepted to evaluate and improve quality of care, as shown by Beck et al. reiterating Codman’s concepts [5]. Three main outcome domains are frequently measured in hip fracture care; (surgical) complications, functional recovery and mortality [6]. Mortality is a measurable and objective parameter. However, neither mortality nor the other outcomes are directly and always related to the hospitals’ performance. Results may be influenced by patient factors such as demographics, functional status and comorbidities, often referred to as ‘Case-mix factors’. Case-mix factors include only characteristics that cannot be influenced by the care provided by the physicians or hospital involved. The case-mix of a hospital reflects its patient demographics and disease burden. If case-mix shows considerable variation between hospitals, development of a case-mix adjustment model is indicated to facilitate a valid hospital comparison.

In April 2016, the Dutch Hip Fracture Audit (DHFA) was implemented to evaluate and improve the care for hip fracture patients in the Netherlands [7]. With use of DHFA data, hospital performances are annually assessed. The quality indicators chosen for the DHFA are in line with the systematic review published by Voeten et al. in which a set of nine quality indicators for hip fracture care was recommended. The set contains mainly structural and process indicators of which several are known to be related to outcomes, however only two direct outcome indicators were recommended; mortality and functional mobility. To date, mortality is not used as a quality indicator in the DHFA as there is no case-mix correction available. This underscores the need for development of a model using case-mix variables that are readily available in the DHFA data. Internationally, the results of this study may be of help in the calibration of other registries; case-mix correction models need to be regularly recalibrated due to the fact that the incidence of 30-day mortality shows a decreasing trend and the profile of hip fracture patients may change over the time [8].

The main objective of this study is to evaluate hospital variation regarding patient demographics and disease burden, to develop a case-mix adjustment model to analyse differences in hip fracture patients’ mortality in order to calculate the case-mix adjusted hospital-specific mortality rate.

## Patients and Methods

Data were derived from the Dutch Hip Fracture Audit (DHFA): a multidisciplinary national registry with a coverage rate of approximately 85% of the 17,500 patients treated annually [1]. All adult hip fracture patients registered between 1–1-2017 and 31–12-2019 were included. Peri-prosthetic and pathological fractures are exclusion criteria for registration in the DHFA. Dates of death were derived from the Dutch Vektis data institute, which collects data from health insurance reimbursements [9]. Data was joined using social security numbers and anonymized by a trusted third party. Patients with missing social security numbers could not be joined and were therefore excluded. No ethical approval for this study type was needed under Dutch law. The main outcomes of this study were 30-day and 90-day mortality defined as mortality within 30 or 90 days after date of admission, respectively.

A selection of potential case-mix factors was made on the basis of expert opinion and availability within the DHFA dataset. The DHFA multidisciplinary scientific committee, consisting of three trauma surgeons, two orthopaedic surgeons, two geriatricians, two internal medicine specialists, one nursing home physician and two clinical researchers, acted as the expert panel. The following potential case-mix factors were selected: Patient characteristics including age, gender, fracture side, fracture type, pre-fracture living situation, Fracture Mobility Score and KATZ Index of Independence in Activities of Daily Living (KATZ-6 ADL) score [10], American Society of Anaesthesiologist physical status classification (ASA-class)[11], pre-fracture diagnosis of dementia or osteoporosis, and nutritional status. Nutritional status was measured using the short nutritional assessment questionnaire (SNAQ) or the malnutrition universal screening tool (MUST) and categorized as low (SNAQ ≤ 1 or MUST 0), medium (SNAQ 2 or MUST 1) or high risk (SNAQ ≥ 3, MUST ≥ 2) [12, 13].

### Statistical analysis

The variation of case-mix factors between hospitals was assessed using logistic regression. In the assessment of between-hospital variation, continuous case-mix factors or factors consisting of multiple categories were categorized, based on the expert’s opinion, as follows: < 80 vs. ≥ 80 years, side left vs. right (bilateral at the same date was excluded here), living at home with or without help vs. living in a nursing home, Fracture Mobility Score ≤ 1 vs. ≥ 2, KATZ6-adl score 0 vs. ≥ 1, ASA-class 1–2 vs. 3–5, risk of malnutrition low vs. medium or high risk. Fracture types were stratified as specific type vs. all other registered fracture types. After dichotomizing each variable, the mean, minimum and maximum percentage over all hospitals were calculated and presented in a violin graph. The significance of this variation was calculated using logistic regression models with case-mix factors as dependent variable and hospitals as independent variable.

The association between 30-day and 90-day mortality and case-mix factors was analysed using multivariable logistic regression models. For these regression models continuous and factor variables were used as registered within the DHFA to optimize the estimation of their effect. Multicollinearity between factors was assessed by calculating variance inflation factors (VIF). Case-mix factors with a VIF > 2.5 were deleted if their attribution was considered to be minimal due to an explainable clinical relation with other factors. A non-linear relation with age was assessed by integrating an age-quadratic term.

Hospital performance regarding mortality was measured as the ratio between the hospital’s observed mortality divided by the expected mortality (O/E ratio) [14]. The unadjusted expected mortality was calculated as the observed mortality rate of all hospitals combined. The adjusted expected mortality per hospital was calculated as the mean predicted probability of survival of the hospital’s patients, which was derived from the multivariable logistic regression model (case-mix model). The observed outcome of a hospital divided by its expected outcome (O/E ratio) indicates their performance: an O/E ratio above 1 indicated that the hospital’s mortality rate was higher than expected, whereas an O/E ratio below 1 indicated that the hospital had a lower mortality rate than expected. The 95% confidence intervals (CI) were calculated to indicate whether the O/E ratio of a hospital was statistically different from that of the other hospitals. When a hospital lies outside this 95%-CI it is seen as a statically significant outlier.

Patients with missing values were analysed as a separate group in the multivariable logistic regression analysis if these exceeded 5% of the total included number of patients. If the number of missing values in a variable was below 5%, the missing patients were excluded from the analysis. Statistical analysis was performed using R Studio Version 1.4.1106 [15].

## Results

A total of 41,212 patients were included, treated in 64 hospitals. The median number of patients included per hospital was 558 (range 20–1,621). 1,838 patients were excluded due to inability to match decease dates to the DHFA data, leaving 39,374 patients eligible for analysis. The overall 30-day mortality was 7.0% (2,757 patients) and overall 90-day mortality was 12.0% (4,735 patients). Baseline characteristics are shown in Supplementary Table 1.

### Between-hospital variation in case-mix factors

**Fig. 1:**
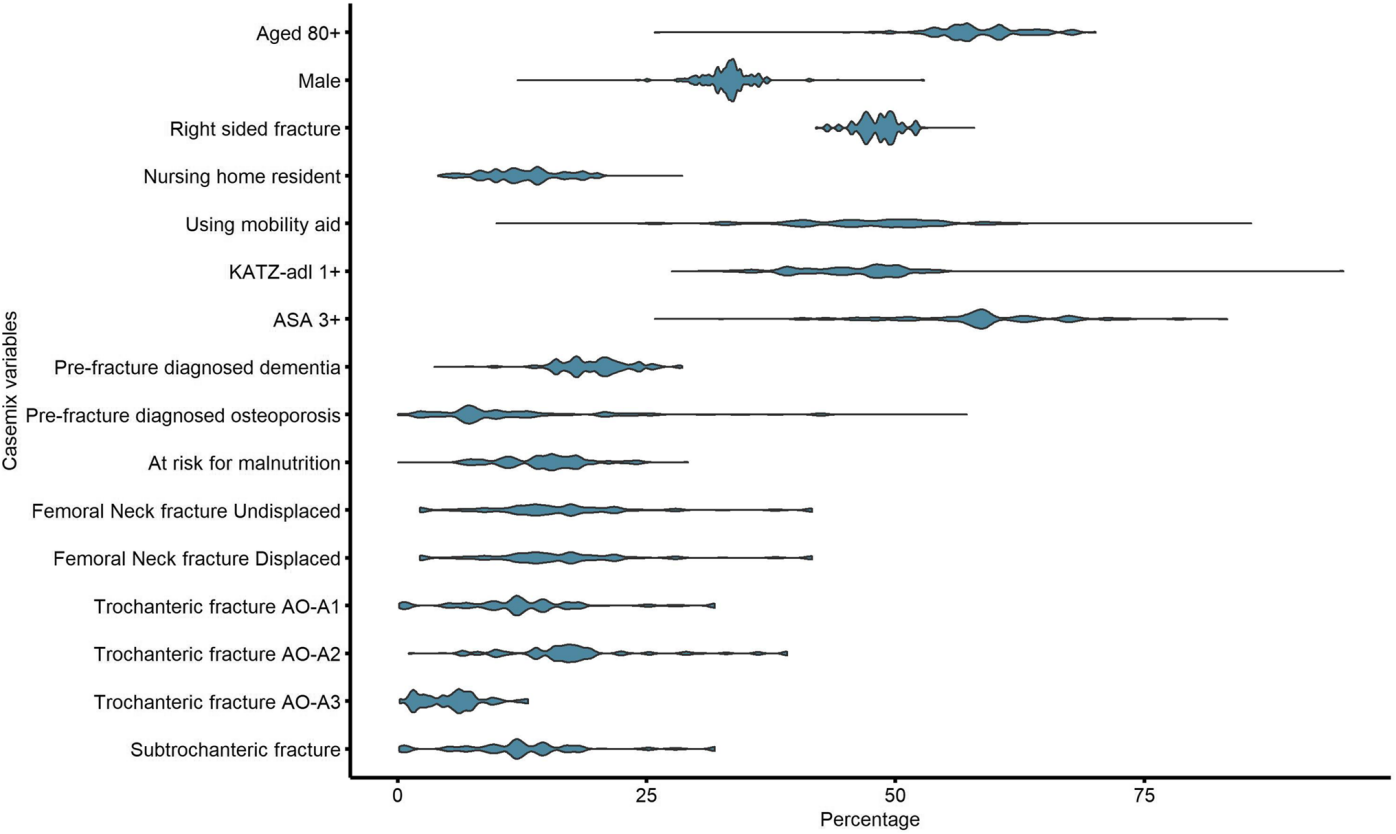
Between hospital variation in case-mix factors

In Fig. 1 and Table 1, the between-hospital variation in case-mix factors is presented. Substantial differences between hospitals’ range and mean percentage of case-mix factors were observed for age ≥ 80 years, male gender, pre-fracture nursing home residents, patients using a mobility aid, KATZ6-adl scores ≥ 1, ASA-class ≥ 3, pre-fracture diagnosed dementia, pre-fracture diagnosed osteoporosis, patients at risk for malnutrition and all fracture types: undisplaced and displaced femoral neck fractures, trochanteric fracture types AO-A1, AO-A2, AO-A3, and subtrochanteric fractures. All of the aforementioned factors had a *p-*value of < 0.001. The only factor with non-significant between-hospital variation was fracture side (42.0–57.9%, *p* = 0,2785).

**Table 1:** Mean percentages (range) of case-mix variables per hospital in the Netherlands

n Hospitals = 64					
Case-mix Factors	Mean %	Min	-	Max %	*p*-value for hospital variation
Age ≥ 80	56.5	25.8	**-**	72.1	< 0.001
Male	33.1	12.0	-	52.9	< 0.001
Right sided fracture	48.3	42.0	**-**	57.9	0.2785
Nursing home resident	11.6	4.0	**-**	28.6	< 0.001
Using mobility aid	46.4	9.9	**-**	85.7	< 0.001
KATZ-adl ≥ 1	45.0	27.5	**-**	95.0	< 0.001
ASA-class ≥ 3	57.4	25.8	**-**	83.3	< 0.001
Pre-fracture diagnosed dementia	18.6	3.6	**-**	28.6	< 0.001
Pre-fracture diagnosed osteoporosis	13.0	0.0	**-**	57.1	< 0.001
At risk for malnutrition	14.9	0.0	**-**	29.2	< 0.001
Femoral Neck fracture Undisplaced	16.4	2.2	**-**	41.6	< 0.001
Femoral Neck fracture Displaced	34.8	0.9	**-**	55.6	< 0.001
Trochanteric fracture AO-A1	12.9	0.1	**-**	31.9	< 0.001
Trochanteric fracture AO-A2	16.5	1.1	**-**	39.1	< 0.001
Trochanteric fracture AO-A3	5.4	0.2	**-**	13.1	< 0.001
Subtrochanteric fracture	3.5	0.0	**-**	10.0	< 0.001

### Case-mix factors for 30-day and 90-day mortality

Several case-mix factors had an independent association with 30-day mortality; age, male gender, all fracture types, higher Pre-Fracture Mobility Scores, Daily living dependency, ASA-class of III or higher, and increased risks of malnutrition. Not statistically significant was fracture side (*p* = 0.90) (Table 2). The analysis for 90-day mortality showed similar results; age, male gender, all fracture types, higher Pre-Fracture Mobility Scores, Daily living dependency, ASA-class of III or higher and increased risks of malnutrition. Not statistically significant was fracture side (*p* = 0.17). The 90-day mortality model is shown in Supplementary Table 2. Multicollinearity was assessed in both models. For both the 30-day and the 90-day mortality model the VIF for the pre-fracture living situation and for dementia were > 2.5. A relation between these factors and the daily living dependency score (KATZ6-adl) and mobility (Fracture Mobility Score) was assumed, therefore pre-fracture living situation and dementia were excluded from the multivariable regression models. After excluding these variables, the VIF was < 2.5 for all variables included.

**Table 2:** Univariable and multivariable logistic regression model to assess the association of patient characteristics with 30-day mortality in hip fracture patients in the Netherlands

		Univariable Analysis					Multivariable Analysis				
Factor	n patients	OR	95%-CI			*p*-value	aOR	95%-CI			*p*-value
Age (per year)	39,260	0.07	1.06	-	1.07	< 0.01	1.06	1.05	-	1.07	< 0.01
Gender						< 0.01					< 0.01
Female	26,268	ref					ref				
Male	13,051	1.82	1.63		2.05	< 0.01	2.00	1.83	-	2.18	< 0.01
Fracture Side						0.10					0.90
Right	18,848	ref					ref				
Left	20,258	0.88	0.79	-	0.99	0.03	0.99	0.91	-	1.08	0.89
Bilateral	28	1.1	0.15	-	8.09	0.93	1.30	0.40	-	4.27	0.67
Fracture Type						0.05					< 0.01
Femoral Neck fracture Undisplaced	6452	ref					ref				
Femoral Neck fracture Displaced	13,517	1.35	1.13	-	1.62	< 0.01	1.29	1.13	-	1.49	< 0.01
Trochanteric fracture AO-A1	5016	1.23	0.98	-	1.54	0.07	1.23	1.04	-	1.45	0.01
Trochanteric fracture AO-A2	6925	1.21	0.98	-	1.49	0.07	1.29	1.10	-	1.50	< 0.01
Trochanteric fracture AO-A3	2078	1.24	0.93	-	1.67	0.15	1.31	1.06	-	1.63	0.01
Subtrochanteric fracture	1200	1.49	1.06	-	2.09	0.02	1.82	1.42	-	2.34	< 0.01
Missing	4186	1.23	0.97	-	1.56	0.08	0.48	0.39	-	0.59	< 0.01
Pre-Fracture Living Situation*						< 0.01					
Independent at home	19,790	ref									
At home with help in daily living	6400	3.35	2.88	-	3.90	< 0.01					
Elderly home	2823	2.81	2.29	-	3.46	< 0.01					
Nursing facility	3944	2.82	2.35	-	3.39	< 0.01					
Revalidation facility	346	2.24	1.27	-	3.94	0.01					
Other	787	2.35	1.62	-	3.42	< 0.01					
Missing	5284	1.76	1.45	-	2.13	< 0.01					
Pre-fracture Mobility Score						< 0.01					< 0.01
Not using any mobility aid	16,474	ref					ref				
Mobile outdoors using 1 mobility aid	2108	2.67	2.05	-	3.46	< 0.01	1.34	1.09	-	1.64	0.01
Mobile outdoors with 2 aids or frame	11,247	2.96	2.53	-	3.48	< 0.01	1.47	1.29	-	1.68	< 0.01
Mobile indoors but never outside without help of others	2784	5.68	4.69	-	6.88	< 0.01	2.66	2.28	-	3.10	< 0.01
No functional mobility (using lower extremities)	972	2.32	1.58	-	3.39	< 0.01	2.72	2.16	-	3.43	< 0.01
Missing	5789	2.62	2.17	-	3.17	< 0.01	1.40	1.18	-	1.66	< 0.01
Daily living dependency						< 0.01					< 0.01
Independent (KATZ6-ADL = 0)	20,129	ref					ref				
Dependent (KATZ6-ADL > 0)	16,819	2.98	2.61	-	3.39	< 0.01	2.27	2.03	-	2.54	< 0.01
Missing	2426	2.18	1.71	-	2.78	< 0.01	1.84	1.50	-	2.25	< 0.01
ASA-class						< 0.01					< 0.01
I and II	14,457	ref					ref				
III, IV and IV	20,291	5.53	4.51	-	6.79	< 0.01	2.64	2.29	-	3.05	< 0.01
Missing	4626	10.02	8.02	-	12.53	< 0.01	10.85	9.16	-	12.86	< 0.01
Pre-fracture diagnosed dementia*						< 0.01					
No	26,960	ref									
Yes	6512	1.96	1.71	-	2.24	< 0.01					
Missing	5902	1.28	1.08	-	1.50	< 0.01					
Pre-fracture diagnosed steoporosis						0.80					< 0.01
No	28,643	ref					ref				
Yes	3898	0.96	0.79	-	1.17	0.70	0.81	0.71	-	0.94	< 0.01
Missing	6833	1.04	0.89	-	1.21	0.64	0.81	0.71	-	0.92	< 0.01
Risk of malnutrition						< 0.01					< 0.01
No risk of malnutrition	30,882	ref					ref				
Slight/medium risk of malnutrition	1424	1.96	1.64	-	2.33	< 0.01	1.41	1.17	-	1.70	< 0.01
High risk of malnutrition	3828	2.75	2.49	-	3.05	< 0.01	1.94	1.74	-	2.17	< 0.01
Missing	3240	1.63	1.43	-	1.86	< 0.01	1.47	1.25	-	1.72	< 0.01

^*^Due to multicollinearity this variable was excluded from the multivariate analysis thereafter all Variance Inflation Factors were < 2,5

The Estimate of the intercept for this model is -9.79. Odds ratios are derived using ${OR= e}^{Estimate}$

### Hospital comparison of 30-day and 90-day mortality

**Fig. 2:**
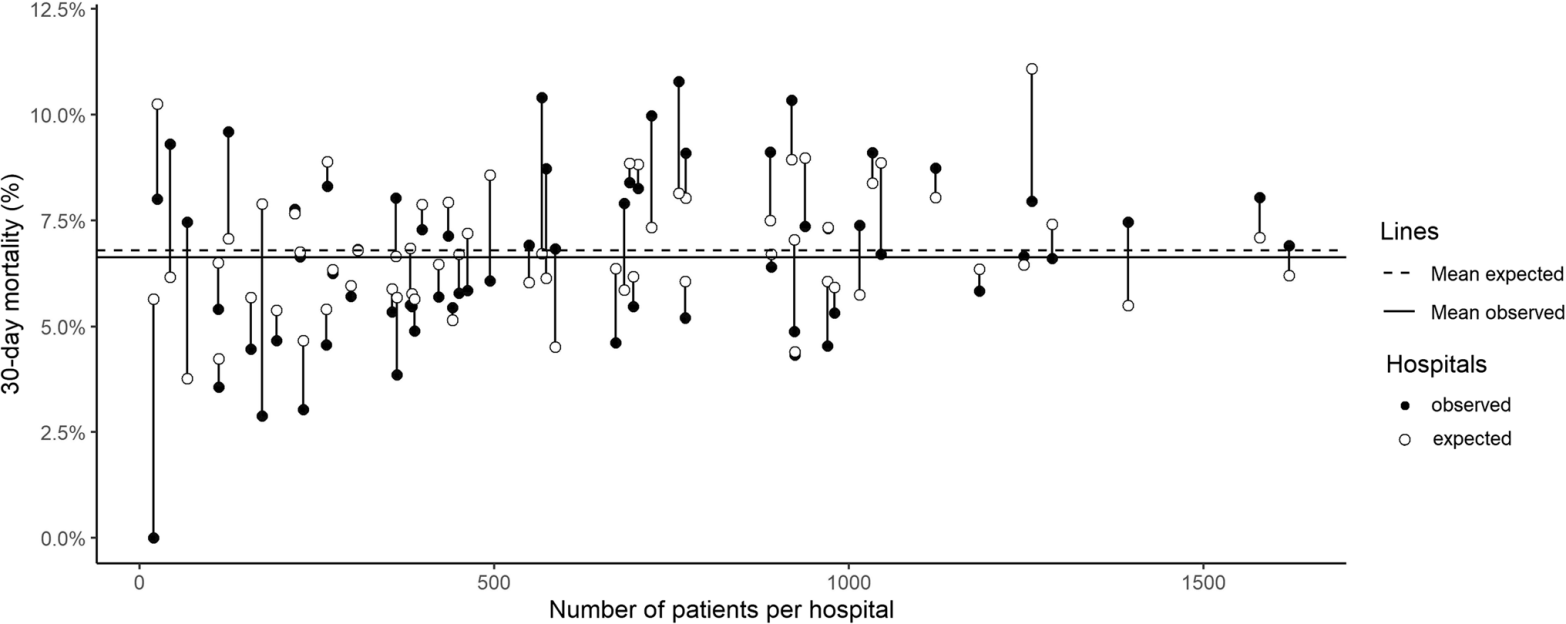
Difference between observed and case-mix expected 30-day mortality in Hip Fracture patients per hospital in the Netherlands

**Fig. 3:**
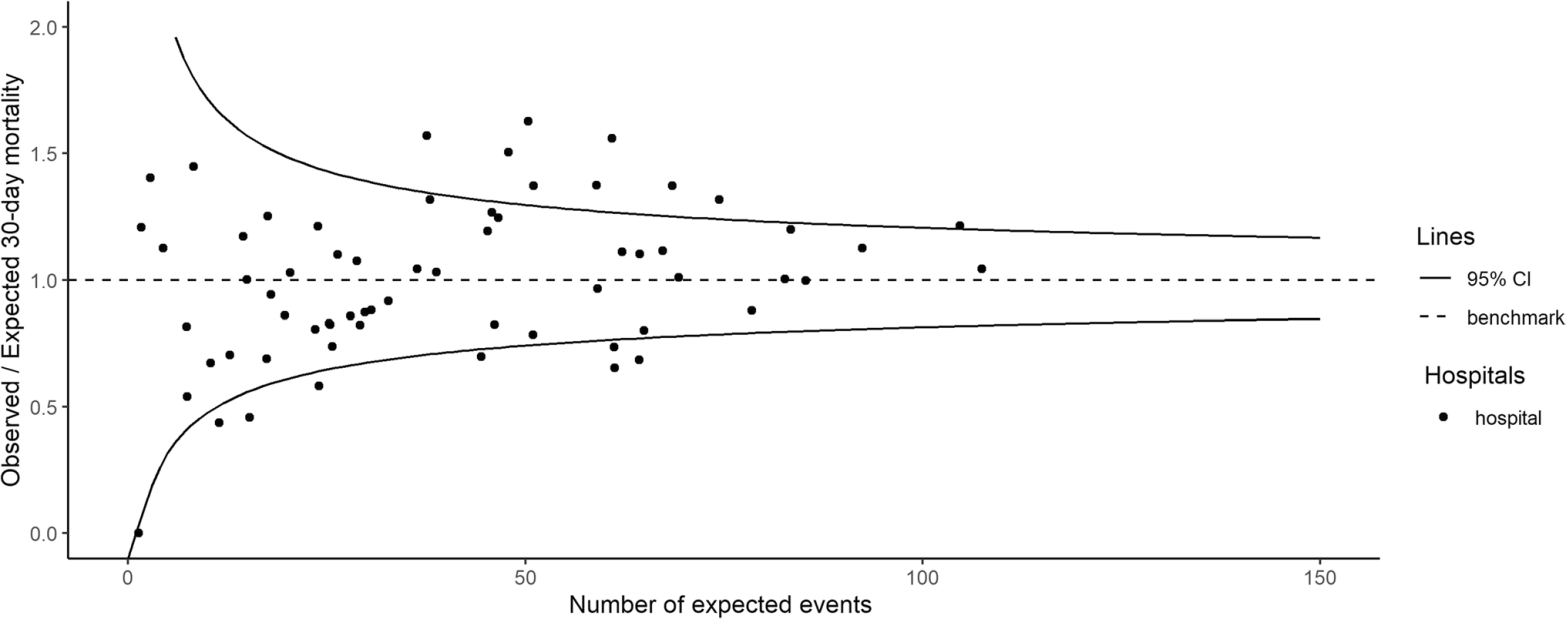
Unadjusted* funnel-plot of between-hospital variation in 30-day mortality in Hip Fracture patients in the Netherlands. The O/E results are shown in funnel-plots in which the volume is shown on the x-axis, the benchmark is shown as a dashed line and the funnel-lines represent the upper and lower limit of the 95%-CI. Hospitals above the 95%-CI funnel-line are considered outliers with statistically significant higher mortality than expected based on their case-mix, hospitals below the 95%-CI line have lower mortality rates than expected. * The expected mortality used for the unadjusted O/E ratio was the average hospital 30-days mortality of 6.6%

**Fig. 4:**
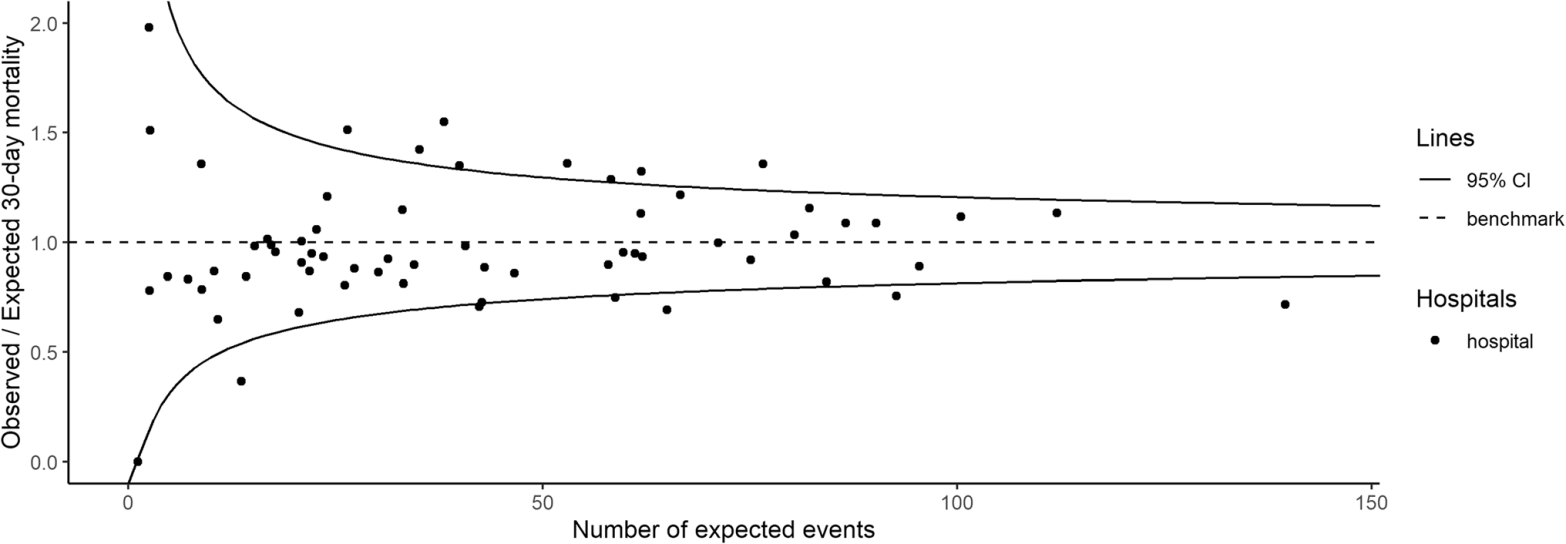
Case-mix adjusted** funnel-plot of between-hospital variation in 30-day mortality in Hip Fracture patients in the Netherlands. The O/E results are shown in funnel-plots in which the volume is shown on the x-axis, the benchmark is shown as a dashed line and the funnel-lines represent the upper and lower limit of the 95%-CI. Hospitals above the 95%-CI funnel-line are considered outliers with statistically significant higher mortality than expected based on their case-mix, hospitals below the 95%-CI line have lower mortality rates than expected. **The expected mortality used for the adjusted O/E ratio was case-mix adjusted for: Age, Gender, Fracture type, Pre Fracture mobility, KATZ6-ADL score, ASA-Class, Osteoporosis and risk of malnutrition

Thirty-day mortality per hospital was on average 6.6% and ranged from 0.0 to 10.8%. Expected 30-day mortality based on the case-mix correction model was on average 6.8%, and ranged from 3.8 to 11.1%. Figure 2 shows for each hospital the difference between observed and expected case-mix-adjusted 30-day mortality. Figure 3 shows that nine hospitals were outliers (outside the 95%-Confidence Interval) with higher than expected mortality rates and eight hospitals were outliers with lower than expected mortality rates without case-mix adjustment. After case-mix correction eight hospitals had statistically significant higher 30-day mortality rates than expected, of which five were other hospitals, and three were the same hospitals as before correction. After case-mix correction six hospitals had statistically significant lower 30-day mortality rates than expected (Fig. 4), of which three were the same hospitals as before correction. The adjusted O/E ratio ranged from 0.0 to 2.0.

Observed 90-day mortality per hospital was on average 11.3% and ranged from 0.0 to 20.9%. Average expected 90-day mortality based on the case-mix correction was 11.7% and ranged from 7.6 to 16.2%. Supplementary  Figure 1 shows the difference between each hospitals’ observed and expected casemix-adjusted 90-day mortality. Supplementary Figure 2 shows eleven hospitals were outliers (outside the 95%-confidence interval) with high mortality rates, and six hospitals were outliers with low mortality rates. After case-mix correction, five of these eleven hospitals remained to be an outlier and one other hospitals became outliers with statistically significant higher 90-day mortality rates than expected. Of the six hospitals with statically significant lower 90-day mortality rates, two remained to be an outlier and five other hospitals became outliers after case-mix correction (Supplementary Figure 3). The adjusted O/E ratio ranged from 0.0 to 2.7.

## Discussion

Significant variation in case-mix factors amongst all participating hospitals in the DHFA was found leading to the conclusion that every hospital treats a different hip fracture population with respect to patient demographics and disease burden. Observed 30-day mortality rates ranged from 0.0% to 10.8 and 90-day mortality rates from 0.0 to 20.9%. Correction for case-mix factors translates to an expected 30-day mortality between 3.8 and 11.1% and 90-day mortality between 7.6 and 16.2%. The average expected mortality rates were slightly higher than observed mortality rates, both at 30-days and 90-days. After correction for case-mix factors significant between-hospital differences (outside 95%-confidence interval) were found regarding 30-day mortality with eight hospitals with higher mortality than expected and six hospitals with lower mortality than expected. Regarding 90-day mortality, six hospitals had higher mortality than expected and seven had lower mortality than expected. Without adjustment there were more outlier hospitals with high mortality rates (above the 95%-confidence interval), which is probably, or at least in part, caused by the case-mix of their patients. Also, several hospitals where shown to be outliers when correcting for case-mix factors. When analysing which specific hospitals were outliers, several hospitals remained outliers regardless of correction, whilst others became outliers, or changed to perform within the 95%-CI. This illustrates the need for case-mix adjustment when comparing hospital performances for hip fracture care.

This study found almost all studied case-mix factors to be associated with mortality at both 30 and 90 days. Most of these case-mix factors were observed to have an independent relationship with mortality in recent systematic reviews [16–19]. Findings are also in line with the case-mix factors used by the English National Hip Fracture Database (NHFD); however, the researchers could not access the exact model currently in use [20].

All case-mix factors with significance had an OR’s greater than 1, corresponding with an increased risk of mortality. The only exception was pre-fracture diagnosed osteoporosis, which had a protective effect after adjustment. The reported pre-fracture diagnosed osteoporosis is highly variable as the between hospital variation ranged from 0.0 to 57.1%. Other case-mix variables also showed wide between-hospital variation, e.g. age ≥ 80 years (25.8–72.1%), male gender (12.0–52.9%). A wide range in mean percentage emphasizes the need for case-mix adjustment on the one hand, but may also be a result of data quality on the other. In some cases, it is more likely that this variability is caused by variability in data quality: especially concerning pre-fracture diagnosed osteoporosis it is likely that the aforementioned protective effect of this osteoporosis variable may be the result of best-practice hospitals performing better at both registration and clinical outcomes. Also, the wide between-hospital variation in several case-mix factors is caused by a few low-volume hospitals of which the smallest included 20 patients. A cut-off value for a minimal number of patients when reporting case-mix adjusted mortality may be appropriate. However, for the purpose of developing a model, the authors decided against it, as determination of the cut-off value would be arbitrary.

Several patient related factors potentially associated with mortality were not included in our study: the presence of specific multiple comorbidities, cardiac diseases, frailty, cancer, renal failure and diabetes. However, we did include the ASA class of patients in the model and ASA class may represent the outline of these comorbidities. Potential case-mix factors found in literature but not included nor comprised within this study are history of delirium and low haemoglobin levels. The evidence of their association with mortality was shown to be moderate, this combined with unavailability of these variables for all patients in the DHFA was the reason for the expert panel not to include them in the case-mix model [19].

The overall 30-day mortality was 7.0% in our study which compares equally to the reported mortality rates of several other national registries with an average of 7.5% [21]. The slightly lower percentage may be due to the lower age and ASA-class of DHFA patients in comparison with other registries [21]. The overall 90-day mortality of 12.0% found in this study also seems in comparable to other registries’ mortality rates, although not all registries report on 90-day mortality. Denmark reported 16% mortality at 90-days, other studies reported 4-months mortality of 12% [21–23]. The lower limit of the range of both 30-day and 90-day mortality observed per hospital was 0.0%, which is caused by a low volume hospital (*n* = 20) in which no patients deceased within 90 days.

Outcomes are needed to be able to reflect on the quality of the process of care [24, 25]. Data on outcome quality indicators for hip fracture patients are hard to collect; only a small proportion of the hip fracture population is seen for their 3 months follow-up consultation, which results in a high risk of selection bias. Not only in the DHFA, but also in other registries the collection of follow-up outcome data appears to be a challenge [4, 26]. When registry data is joined with decease dates from trustable data sources, case-mix adjusted mortality data become relatively easily collectable and will serve as an objective parameter for hospital comparisons. As shown in Fig. 4 and Supplementary Figure 3, the mortality rates of participating hospitals still differed significantly after case-mix adjustment. When assuming the case-mix correction to be correct and complete these statistical differences in mortality may be due to the quality of care provided by the outlier hospitals. In order to improve of hip fracture care nationwide positive outliers could serve as a best practice examples while negative outliers may learn from others by reflection on their own process of care, resulting in better overall care.

This study has several limitations. First of all, working with registry-data implies that data quality depends on the quality of registration by hospitals. Also, several variables are not registered in the DHFA, such as medical history, comorbidities, concomitant injuries and trauma mechanism. The latter however—the factor severe trauma—is thought to be of small impact on case-mix models as less than 0.1% of all hip fracture patients had an Injury Severity Score ≥ 16 in the Netherlands over the past years [1]. Also, mortality rates in our data did not differ significantly when comparing level I trauma centers to the non-trauma center hospitals. Secondly, the registry data used is not validated by the researchers and there is no possibility to complement missing values. Due to the missing of social service numbers 4.5% of the population (*n* = 1,838) had to be excluded because joining data from the DHFA with Vektis data was not possible. However, a missing data analysis showed these numbers to be missing at random and therefore they are assumed to not have resulted in selection bias. There were several case-mix factors for which > 5% of patients had missing values, of which the included ‘missing’ categories had high OR’s in both models. Multiple imputation was considered; however, this model is intended to be used on real time registration data in which patients’ case-mix factors are likely to not always be complete. Also, the missing data in case-mix factors might not be missing completely at random, therefore including ‘missing’ as category for several case-mix factors improves the accuracy of this case-mix correction model. This directly leads to the strength of this study: it describes a case-mix model applicable for real-life data based on a large number of patients.

In the future, after a prolonged registration period and improved and validated data quality perhaps internal validation is possible, as well the improvement of this model by adding new case-mix variables and development of case-mix models for other outcomes such as functional mobility and in-hospital complications.

## Conclusion

This study showed a significant between-hospital variation in case-mix of hip fracture patients within the Netherlands, as well as a wide between-hospital variation in observed 30-day mortality and 90-day mortality. After adjusting for case-mix with this model mortality rates still differed significantly with both positive and negative outlier hospitals, of which several were other hospitals than before correction. Analysis of outlier hospitals may serve as a starting point for targeted improvement of hip fracture care delivered within the Netherlands. These findings emphasize the importance of adjustment for patient demographics and disease burden when comparing hospitals performances in hip fracture care.

## Supplementary Information

Below is the link to the electronic supplementary material.

Supplementary file1 (Difference between observed and case-mix expected 90-day mortality in Hip Fracture patients in the per hospital in the Netherlands. JPEG 234 KB)

Supplementary file2 (Unadjusted* funnel-plot of between-hospital variation in 90-day mortality in Hip Fracture patients in the Netherlands. The O/E results are shown in funnel-plots in which the volume is shown on the x-axis, the benchmark is shown as a dashed line and the funnel-lines represent the upper and lower limit of the 95%-CI. Hospitals above the 95%-CI funnel-line are considered outliers with statistically significant higher mortality than expected based on their case-mix, hospitals below the 95%-CI line have lower mortality rates than expected. * The expected mortality used for the unadjusted O/E ratio was the average hospital 90-days mortality of 11.3%. JPEG 201 KB)

Supplementary file3 (Case-mix adjusted** funnel-plot of between-hospital variation in 90-day mortality in Hip Fracture patients in the Netherlands. The O/E results are shown in funnel-plots in which the volume is shown on the x-axis, the benchmark is shown as a dashed line and the funnel-lines represent the upper and lower limit of the 95%-CI. Hospitals above the 95%-CI funnel-line are considered outliers with statistically significant higher mortality than expected based on their case-mix, hospitals below the 95%-CI line have lower mortality rates than expected. ** The expected mortality used for the adjusted O/E ratio was Case-mix adjusted for: Age, Gender, Fracture type, Pre Fracture mobility, KATZ6-ADL score, ASA-Class, Osteoporosis and risk of malnutrition. JPEG 142 KB)

Supplementary file4 (DOCX 23.5 KB)

## Declarations

### Collaborators

The Dutch Hip Fracture Audit Group: AJ Arends, AH Calf, PW van Egmond, M van Eijk, MJ Heetveld, M van Heijl, MC Luyten, BC van Munster, BG Schutte, SC Voeten.
