# Correction to: Development of a data-driven case-mix adjustment model for comparison of hospital performance in hip fracture care

**DOI:** 10.1007/s11657-022-01121-w

**Published:** 2022-07-19

**Authors:** Franka S. Würdemann, Arthur K. E. Elfrink, Janneke A. Wilschut, Crispijn L. van den Brand, Inger B. Schipper, Johannes H. Hegeman

**Affiliations:** 1 Dutch Institute for Clinical Auditing, Scientific Bureau, Rijnsburgerweg 10, AA, 2333 Leiden, The Netherlands; 2 Department of Traumasurgery, Leiden University Medical Center, Leiden Albinusdreef 2, Leiden, 2333 ZA The Netherlands; 3 Department of Surgery, University Medical Center, Groningen Hanzeplein 1, GZ, 9713 Groningen, The Netherlands; 4 Department of Traumasurgery, Ziekenhuisgroep Twente, Zilvermeeuw 1, Almelo, 7609, PP The Netherlands

**Correction to** **:** **Archives of Osteoporosis (2022) 17:1-10**

**https://doi.org/10.1007/s11657-022-01094-w**

In this article, The Dutch Hip Fracture Audit Group: AJ Arends, AH Calf, PW van Egmond, M van Eijk, MJ Heetveld, M van Heijl, MC Luyten, BC van Munster, BG Schutte, and SC Voeten was missing from the collaborators’ list.

In this article, the “Competing interests” statement and the “Ethical approval” statement were missing and should have been the “Competing interests” statement: Each author certifies that he or she has no commercial associations that might pose a conflict of interest in connection with the submitted article.

Ethical approval

Permission for the use of pseudonymized patient data for research purposes is assured within the Dutch Hip Fracture Audit. Due to the nature of this study, no patient informed consent or approval of the medical ethical commission was required. The original article has been corrected.

