# Supplementary figures and images for: Development of a data-driven case-mix adjustment model for comparison of hospital performance in hip fracture care
Source: Arch Osteoporos. 2022 Apr 27;17(1):73. doi: 10.1007/s11657-022-01094-w (PMC9046354; doi:10.1007/s11657-022-01094-w)

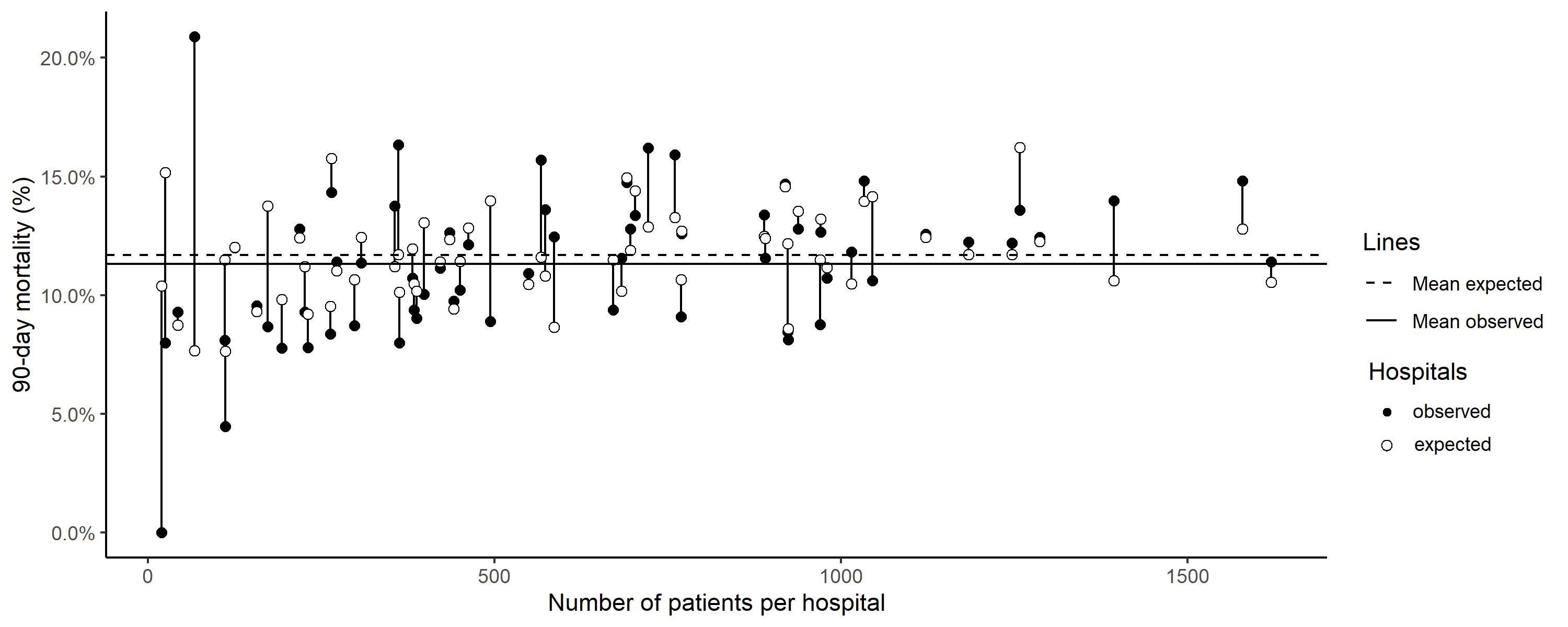

Supplement: Supplementary file 1 — Supplementary file1 (Difference between observed and case-mix expected 90-day mortality in Hip Fracture patients in the per hospital in the Netherlands. JPEG 234 KB) [file 11657_2022_1094_MOESM1_ESM.jpeg]

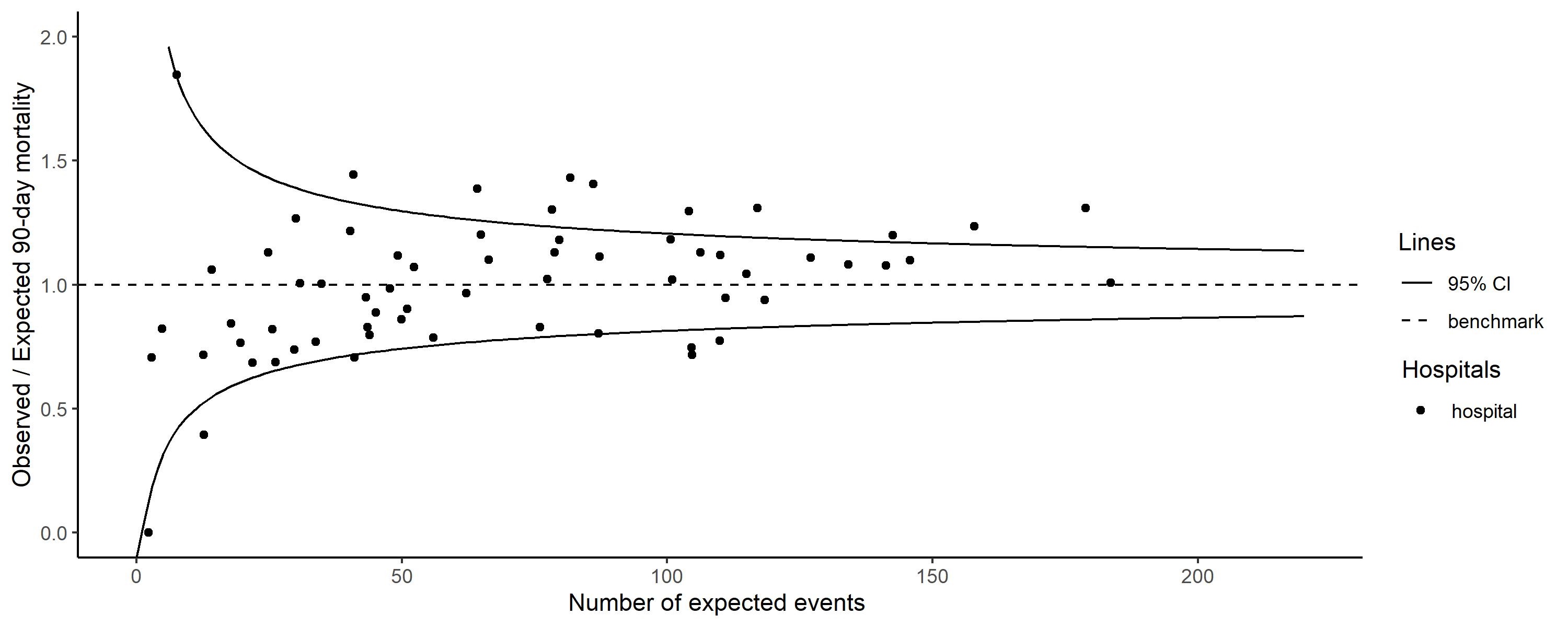

Supplement: Supplementary file 2 — Supplementary file2 (Unadjusted* funnel-plot of between-hospital variation in 90-day mortality in Hip Fracture patients in the Netherlands. The O/E results are shown in funnel-plots in which the volume is shown on the x-axis, the benchmark is shown as a dashed line and the funnel-lines represent the upper and lower limit of the 95%-CI. Hospitals above the 95%-CI funnel-line are considered outliers with statistically significant higher mortality than expected based on their case-mix, hospitals below the 95%-CI line have lower mortality rates than expected. * The expected mortality used for the unadjusted O/E ratio was the average hospital 90-days mortality of 11.3%. JPEG 201 KB) [file 11657_2022_1094_MOESM2_ESM.jpeg]

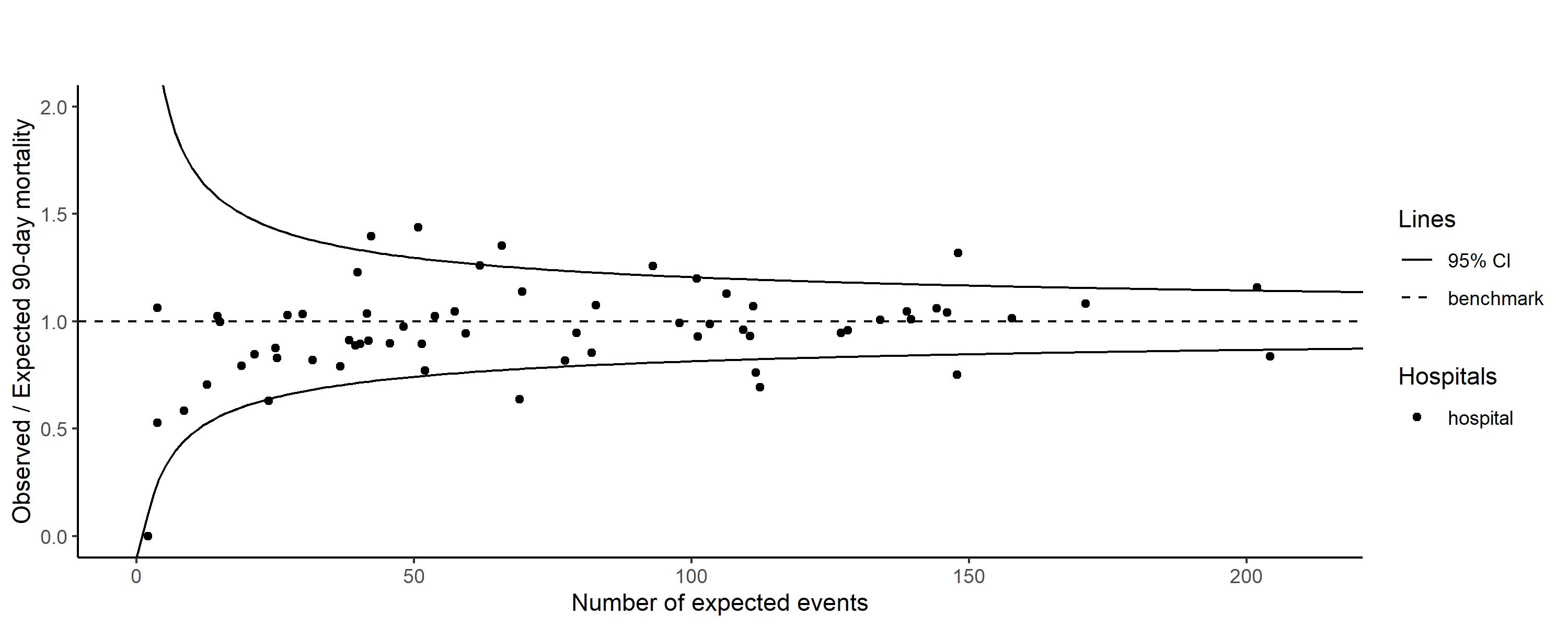

Supplement: Supplementary file 3 — Supplementary file3 (Case-mix adjusted** funnel-plot of between-hospital variation in 90-day mortality in Hip Fracture patients in the Netherlands. The O/E results are shown in funnel-plots in which the volume is shown on the x-axis, the benchmark is shown as a dashed line and the funnel-lines represent the upper and lower limit of the 95%-CI. Hospitals above the 95%-CI funnel-line are considered outliers with statistically significant higher mortality than expected based on their case-mix, hospitals below the 95%-CI line have lower mortality rates than expected. ** The expected mortality used for the adjusted O/E ratio was Case-mix adjusted for: Age, Gender, Fracture type, Pre Fracture mobility, KATZ6-ADL score, ASA-Class, Osteoporosis and risk of malnutrition. JPEG 142 KB) [file 11657_2022_1094_MOESM3_ESM.jpeg]
