# Supplementary material for: Development of a data-driven case-mix adjustment model for comparison of hospital performance in hip fracture care
Source: Arch Osteoporos. 2022 Apr 27;17(1):73. doi: 10.1007/s11657-022-01094-w (PMC9046354; doi:10.1007/s11657-022-01094-w)
Supplement: Supplementary file 4 — Supplementary file4 (DOCX 23.5 KB) [file 11657_2022_1094_MOESM4_ESM.docx]

| **Supplementary Table 1. Baseline characteristics of patients with a Hip Fracture in the Netherlands between 2017 and 2019** | | |
| --- | --- | --- |
|  |  | n (%) |
| Total number of patients | | 39374 |
|  |  |  |
| Age (mean (SD)) | | 79.39 (12.07) |
| Gender (%) | |  |
|  | Male | 13051 (33.1) |
|  | Female | 26268 (66.7) |
|  | Missing | 55 (0.1) |
| Fracture Side (%) | |  |
|  | Right | 18848 (47.9) |
|  | Left | 20258 (51.5) |
|  | Bilateral | 28 (0.1) |
|  | Missing | 240 (0.6) |
| Fracture Type (%) | |  |
|  | Femoral Neck fracture Undisplaced | 6452 (16.4) |
|  | Femoral Neck fracture Displaced | 13517 (34.3) |
|  | Trochanteric fracture AO-A1 | 5016 (12.7) |
|  | Trochanteric fracture AO-A2 | 6925 (17.6) |
|  | Trochanteric fracture AO-A3 | 2078 (5.3) |
|  | Subtrochanteric fracture | 1200 (3.0) |
|  | Unspecified | 849 (2.2) |
|  | Missing | 3337 (8.5) |
| ASA-Class | |  |
|  | 1 and 2 | 14457 (36.7) |
|  | 3,4 and 5 | 20291 (51.5) |
|  | Missing | 4626 (11.7) |
| Pre-Fracture Living Situation (%) | |  |
|  | Independent at home | 19790 (50.3) |
|  | At home with help in daily living | 6400 (16.3) |
|  | Elderly home | 2823 (7.2) |
|  | Nursing facility | 3944 (10.0) |
|  | Revalidation facility | 346 (0.9) |
|  | Other | 787 (2.0) |
|  | Missing | 5284 (13.4) |
| Pre-fracture Mobility Score (%) | |  |
|  | Unknown | 2471 (6.3) |
|  | Not using any mobility aid | 16474 (41.8) |
|  | Mobile outdoors using 1 mobility aid | 2108 (5.4) |
|  | Mobile outdoors with 2 aids or frame | 11247 (28.6) |
|  | Mobile indoors but never outside without help of others | 2784 (7.1) |
|  | No functional mobility (using lower extremities) | 972 (2.5) |
|  | Missing | 3318 (8.4) |
| Daily Living Dependency (%) | |  |
|  | Independent (KATZ6-ADL = 0) | 20129 (51.1) |
|  | Dependent (KATZ6-ADL > 0) | 16819 (42.7) |
|  | Missing | 2426 (6.2) |
| Pre-fracture diagnosed dementia (%) | |  |
|  | No | 26960 (68.5) |
|  | Yes | 6512 (16.5) |
|  | Missing | 5902 (15.0) |
| Pe-fracture diagnosed osteoporosis (%) | |  |
|  | No | 28643 (72.7) |
|  | Yes | 3898 (9.9) |
|  | Missing | 6833 (17.4) |
| Risk of malnutrition (%) | |  |
|  | No risk of malnutrition | 30882 (78.4) |
|  | Slight/medium risk of malnutrition | 1424 (3.6) |
|  | High risk of malnutrition | 3828 (9.7) |
|  | Missing | 3240 (8.2) |

| **Supplementary Table 2. Univariable and multivariable logistic regression model to assess the association of patient characteristics with 90-day mortality in hip fracture patients in the Netherlands** | | | | | | | | | | | | | | |
| --- | --- | --- | --- | --- | --- | --- | --- | --- | --- | --- | --- | --- | --- | --- |
|  |  |  |  | Univariate Analysis | | | | |  | Multivariate Analysis | | | | |
| Factor | | n patients |  | OR | 95%-CI | | | *p*-value |  | aOR | 95%-CI | | | *p*-value |
| Age |  | 39260 |  | 1.08 | 1.08 | - | 1.08 | <0.01 |  | 1.06 | 1.05 | - | 1.06 | <0.01 |
| Gender | |  |  |  |  |  |  | <0.01 |  |  |  |  |  | <0.01 |
|  | Female | 26268 |  | ref |  |  |  |  |  | ref |  |  |  |  |
|  | Male | 13051 |  | 1.38 | 1.29 | - | 1.46 | <0.01 |  | 1.97 | 1.83 | - | 2.11 | <0.01 |
| Fracture Side | |  |  |  |  |  |  | <0.01 |  |  |  |  |  | 0.17 |
|  | Right | 18848 |  | ref |  |  |  |  |  | ref |  |  |  |  |
|  | Left | 20258 |  | 0.9 | 0.85 | - | 0.95 | <0.01 |  | 0.94 | 0.88 | - | 1.00 | 0.06 |
|  | Bilateral | 28 |  | 1.42 | 0.54 | - | 3.73 | 0.48 |  | 0.82 | 0.26 | - | 2.65 | 0.75 |
| Fracture Type | |  |  |  |  |  |  | <0.01 |  |  |  |  |  | <0.01 |
|  | Femoral Neck fracture Undisplaced | 6452 |  | ref |  |  |  |  |  | ref |  |  |  |  |
|  | Femoral Neck fracture Displaced | 13517 |  | 1.38 | 1.25 | - | 1.52 | <0.01 |  | 1.19 | 1.07 | - | 1.33 | <0.01 |
|  | Trochanteric fracture AO-A1 | 5016 |  | 1.47 | 1.31 | - | 1.65 | <0.01 |  | 1.17 | 1.03 | - | 1.34 | 0.01 |
|  | Trochanteric fracture AO-A2 | 6925 |  | 1.54 | 1.38 | - | 1.71 | <0.01 |  | 1.18 | 1.05 | - | 1.33 | 0.01 |
|  | Trochanteric fracture AO-A3 | 2078 |  | 1.41 | 1.21 | - | 1.64 | <0.01 |  | 1.20 | 1.02 | - | 1.42 | 0.03 |
|  | Subtrochanteric fracture | 1200 |  | 1.59 | 1.32 | - | 1.90 | <0.01 |  | 1.50 | 1.22 | - | 1.84 | <0.01 |
|  | Missing | 4186 |  | 1.28 | 1.13 | - | 1.45 | <0.01 |  | 0.61 | 0.52 | - | 0.71 | <0.01 |
| Pre-Fracture Living Situation* | |  |  |  |  |  |  | <0.01 |  |  |  |  |  |  |
|  | Independent at home | 19790 |  | ref |  |  |  |  |  |  |  |  |  |  |
|  | At home with help in daily living | 6400 |  | 3.9 | 3.57 | - | 4.26 | <0.01 |  |  |  |  |  |  |
|  | Elderly home | 2823 |  | 5.57 | 5.01 | - | 6.19 | <0.01 |  |  |  |  |  |  |
|  | Nursing facility | 3944 |  | 8.22 | 7.5 | - | 9,00 | <0.01 |  |  |  |  |  |  |
|  | Revalidation facility | 346 |  | 3.21 | 2.38 | - | 4.31 | <0.01 |  |  |  |  |  |  |
|  | Other | 787 |  | 3.88 | 3.21 | - | 4.70 | <0.01 |  |  |  |  |  |  |
|  | Missing | 5284 |  | 2.19 | 1.97 | - | 2.43 | <0.01 |  |  |  |  |  |  |
| Pre-fracture Mobility Score | |  |  |  |  |  |  | <0.01 |  |  |  |  |  | <0.01 |
|  | Not using any mobility aid | 16474 |  | ref |  |  |  |  |  | ref |  |  |  |  |
|  | Mobile outdoors using 1 mobility aid | 2108 |  | 2.92 | 2.54 | - | 3.36 | <0.01 |  | 1.31 | 1.12 | - | 1.53 | <0.01 |
|  | Mobile outdoors with 2 aids or frame | 11247 |  | 3.91 | 3.59 | - | 4.25 | <0.01 |  | 1.49 | 1.35 | - | 1.64 | <0.01 |
|  | Mobile indoors but never outside without help of others | 2784 |  | 7.01 | 6.30 | - | 7.81 | <0.01 |  | 2.29 | 2.02 | - | 2.59 | <0.01 |
|  | No functional mobility (using lower extremities) | 972 |  | 4.69 | 3.96 | - | 5.56 | <0.01 |  | 2.34 | 1.93 | - | 2.84 | <0.01 |
|  | Missing | 5789 |  | 2.99 | 2.71 | - | 3.31 | <0.01 |  | 1.41 | 1.23 | - | 1.60 | <0.01 |
| Daily living dependency | |  |  |  |  |  |  | <0.01 |  |  |  |  |  | <0.01 |
|  | Independent (KATZ6-ADL = 0) | 20129 |  | ref |  |  |  |  |  | ref |  |  |  |  |
|  | Dependent (KATZ6-ADL > 0) | 16819 |  | 4.83 | 4.05 | - | 5.19 | <0.01 |  | 2.24 | 2.06 | - | 2.43 | <0.01 |
|  | Missing | 2426 |  | 2.77 | 2.43 | - | 3.16 | <0.01 |  | 1.69 | 1.44 | - | 2.00 | <0.01 |
| ASA-class | |  |  |  |  |  |  | <0.01 |  |  |  |  |  | <0.01 |
|  | I and II | 14457 |  | ref |  |  |  |  |  | ref |  |  |  |  |
|  | III, IV and IV | 20291 |  | 5.38 | 4.90 | - | 5.91 | <0.01 |  | 2.66 | 2.40 | - | 2.95 | <0.01 |
|  | Missing | 4626 |  | 7.28 | 6.52 | - | 8.14 | <0.01 |  | 6.34 | 5.53 | - | 7.26 | <0.01 |
| Pre-fracture diagnosed dementia* | |  |  |  |  |  |  | <0.01 |  |  |  |  |  |  |
|  | No | 26960 |  | ref |  |  |  |  |  |  |  |  |  |  |
|  | Yes | 6512 |  | 4.17 | 3.90 | - | 4.47 | <0.01 |  |  |  |  |  |  |
|  | Missing | 5902 |  | 1.44 | 1.32 | - | 1.57 | <0.01 |  |  |  |  |  |  |
| Pre-fracture diagnosed osteoporosis | |  |  |  |  |  |  | 0.40 |  |  |  |  |  | <0.01 |
|  | No | 28643 |  | ref |  |  |  |  |  | ref |  |  |  |  |
|  | Yes | 3898 |  | 1.04 | 0.94 | - | 1.15 | 0.40 |  | 0.81 | 0.72 | - | 0.90 | <0.01 |
|  | Missing | 6833 |  | 1.05 | 0.97 | - | 1.13 | 0.24 |  | 0.85 | 0.77 | - | 0.95 | <0.01 |
| Risk of malnutrition | |  |  |  |  |  |  | <0.01 |  |  |  |  |  | <0.01 |
|  | No risk of malnutrition | 30882 |  | ref |  |  |  |  |  | ref |  |  |  |  |
|  | Slight/medium risk of malnutrition | 1424 |  | 2.28 | 1.99 |  | 2.61 | <0.01 |  | 1.68 | 1.45 | - | 1.94 | <0.01 |
|  | High risk of malnutrition | 3828 |  | 2.92 | 2.69 |  | 3.17 | <0.01 |  | 2.13 | 1.95 | - | 2.33 | <0.01 |
|  | Missing | 3240 |  | 1.48 | 1.33 |  | 1.65 | <0.01 |  | 1.41 | 1.24 | - | 1.61 | <0.01 |
| *Due to multicollinearity this variable was excluded from the multivariate analysis thereafter all Variance Inflation Factors were < 2,5  The Estimate of the intercept for this model is -8.89. Odds ratios are derived using ${OR= e}^{Estimate}$ | | | | | | | | | | | | | | |
